# General practice consultations, diagnostic investigations, and prescriptions in the year preceding a lung cancer diagnosis

**DOI:** 10.1002/cam4.965

**Published:** 2016-11-23

**Authors:** Louise M. Guldbrandt, Henrik Møller, Erik Jakobsen, Peter Vedsted

**Affiliations:** ^1^ Research Centre for Cancer Diagnosis in Primary CareResearch Unit for General PracticeAarhus UniversityDenmark; ^2^ King's College LondonLondonUnited Kingdom; ^3^ Department of Thoracic SurgeryOdense University HospitalDenmark

**Keywords:** COPD, diagnosis, early diagnosis, general practice, lung cancer

## Abstract

Patterns of general practice utilization in the period before lung cancer (LC) diagnosis may provide new knowledge to ensure timelier and earlier diagnosis of LC. This study aimed to explore the prediagnostic activity in general practice in the year preceding LC diagnosis. The activity was compared to a matched comparison group. We compared LC patients with different stage, and patients with and without chronic obstructive pulmonary disease (COPD). Using Danish registers, we performed a population‐based matched cohort study including lung cancer patients (*n* = 34,017) and matched comparison subjects (*n* = 340,170). During months 12 to 1 prior to diagnosis, 92.6% of LC patients and 88.4% of comparison subjects had one or more contacts with general practice. 13.0% of LC patients and 3.3% of comparison subjects had two or more X‐rays. 20.8% of LC patients and 8.5% of comparison subjects had two or more first‐time antibiotics prescriptions. The incidence rate ratio for having a contact to general practice was similar for LC patients with localized disease compared to LC patients with metastatic disease. LC patients with COPD had more frequent contacts, lung functions tests, X‐rays, and prescriptions than COPD patients without lung cancer, but not as pronounced as compared to patients without COPD. There was a significant increase in healthcare seeking and diagnostic activity in the year prior to a LC diagnosis, regardless of stage at diagnosis. COPD may mask the symptoms of LC. This indicates the presence of a “diagnostic time window” and a potential for more timely diagnosis of LC based on clinical signs and symptoms.

## Introduction

Lung cancer is a significant health problem worldwide and the most common cause of cancer death in the industrialized world 1. In Denmark, lung cancer comprises 12% of all new cancer cases 2. Survival from lung cancer is related to the stage of disease, and 5‐year survival is 50% for localized lung cancer and 2% for a lung cancer with distant spread. Earlier diagnosis of lung cancer may be beneficial in allowing more lung cancer patients curative treatment. Danish lung cancer patients have lower survival than patients from comparable European countries 3, 4. This can possibly be explained by later diagnosis of lung cancer in Denmark and research indicates a lower proportion of lung cancer patients in curable stage in Denmark compared to Norway and Sweden 5. Thus, it is possible that the survival deficit in Danish patients may relate to processes of cancer awareness and diagnostic activity at the level of primary care. Delay in diagnosis before admission to a hospital can be due to either patients delaying going to the general practitioner (GP) (the so called patient's interval) or the GP delaying referral (doctor's interval). This warrants scrutiny of the diagnostic pathway in Danish lung cancer patients.

Most lung cancer patients present with a range of symptoms to the GP in the months before diagnosis 6, 7, 8, 9. The symptoms, such as cough and breathlessness, are relatively common in the general population 10, and alarm symptoms for lung cancer have low‐positive predictive values 11. Symptoms and signs from lung cancer can mimic common diseases such as chronic obstructive pulmonary disease (COPD), leading to a risk for delayed diagnosis 12.

Only a third of lung cancer patients present to GPs with an alarm symptom (such as prolonged coughing, hemoptysis, or weight loss) 12 which is the entrance criterion for the urgent referral route for lung cancer. The PPVs for such symptoms are, depending on the patient's age, between 1% and 4.5%.

Consequently, only 25% of all lung cancer patients are diagnosed through this route 12, 13. There seems to be a need for other referral options for this group of patients. For persons who were later diagnosed with lung cancer we quantified the prediagnostic activity in general practice along with a matched comparison group, and we compared the activity between lung cancer patients with different stage, and between lung cancer patients with and without COPD.

## Methods

### Study design and study population

In Denmark (5.6 million inhabitants), 4560 new lung cancer cases are diagnosed yearly. The age‐standardized (World standard population) lung cancer incidence rates for men and women were 49.9 and 36.7, respectively, with a 1.4% decrease for men and 0.1% increase for women during the study period. We performed a population‐based matched cohort study using register data. Information was collected from nationwide registers at individual level using the unique civil registration number assigned to all Danish residents 14. In Denmark, GPs are gatekeepers to the rest of the health care system and 98% of citizens are listed with a general practice and have free access. GPs are remunerated based on a mixed capitation and fee‐for‐service (25/75%) which ensures complete registration of their services.

#### Lung cancer patients

Patients diagnosed with lung cancer (according to the International Classification of Diseases, ICD‐10 C34) from 1 January 2003 to 31 December 2012 (*n* = 36,342) in Denmark were identified in the Danish Cancer Registry (DCR) and the Danish Lung Cancer Registry (DLCR). The DCR is a national register of all Danish cancer patients and holds information on date of diagnosis (ICD‐10), anatomical site, morphology, and stage. If a patient develops more than one primary cancer, each cancer is registered as a separate record. In the DCR, information about tumor stage at diagnosis is provided by a multidisciplinary team decision, it contains both cTNM and pTNM if available. Reporting to the DCR became mandatory in 1987 15. The DLCR was established in 2001. It contains clinical information about Danish lung cancer patients such as lung function, smoking history, and stage, which are combined with data on cancer treatment and follow‐up. In the DLCR, information about tumor stage at diagnosis is provided by a multidisciplinary team decision with one TNM stage (which can be either cTNM or pTNM). Since 2003, the DLCR includes data on more than 90% of all lung cancer cases in Denmark 16.

Lung cancer patients were excluded from the analyses if they had an incorrect civil registration number (*n* = 398 [1.1%]), had lived outside Denmark at some point during the 12 months preceding the diagnosis (*n* = 670 [1.8%]) or were not listed with a general practice (*n* = 672 [1.8%]). We furthermore excluded 163 (0.5%) patients aged less than 40 years and 202 (0.6%) patients aged more than 90 years.

#### The comparison cohort

Ten comparison subjects were selected for each patient. They matched the lung cancer patient on year of birth, gender and were listed with the same general practice as the lung cancer patients at time of diagnosis (index date). As for patients, these persons were ineligible if they had been living outside Denmark at some point during the 12 months before the index date. Patients and comparison subjects could have no record of any cancer in the DCR at the time of the index date (except nonmelanoma skin cancer).

We excluded 220 (0.6%) lung cancer patients which we were not able to match with comparison subjects. This left 34,017 lung cancer patients for analysis.

### Prediagnostic activity

In order to estimate the prediagnostic health care activity for patients, we selected a range of diagnostic test or treatments made in general practice that a GP would use if a patient presented with pulmonary symptoms.

### Data sources

Information about general practice enrolment was available from the Patient List Register, which is an administrative database that holds information on which general practice each person is listed with at any given time.

#### Activity in general practice

The Danish National Health Service Registry (HSR) was used to gather information about contacts in general practice 17. Face‐to‐face consultations, home visits and telephone contacts during daytime were included. HSR was also used to obtain information about numbers and dates of lung function tests performed in general practice. The tests included: extended lung function test with spirometry (activity code: 7113), double lung function test for exercise‐induced asthma (7121) and peak flow (7183).

The prescriptions of medicines for respiratory diseases and infections were collected from the Danish National Prescription Registry (DNPR) 18. The products included: adrenergic (ATC: R03AC, R03CC), adrenergic and glucocorticoids (RO3AK) anticholinergics (R03BB), theophylline (R03DA), glucocorticoids, systemic (H02AB), leukotrienes (R03DC), glucocorticoids, inhalation (R03BA), penicillin (J01C), and tetracycline (J01AA). These medicines are all available by prescription only.

In the analyses we focused on new or first‐time prescriptions redeemed and lung function test performed in the 12 months before diagnosis; that is, that it was only included if there had been no prescription redeemed or lung function test performed in the period between 12 and 36 months before lung cancer diagnosis.

Data on radiology procedures were obtained from the HSR and the Danish National Patient Registry (NPR) 19. The NPR is a national population‐based database containing admission and discharge dates, combined with diagnoses classified according to ICD‐10.

#### COPD diagnoses

We identified patients as having COPD if they in the period 12–36 months before lung cancer diagnosis had either at least two redeemed prescriptions of relevant medicine, an inpatient visit (See appendix, Table 1, for the ICD10 codes for the inpatient visit) or at least two lung function tests performed in general practice 20. The medicine included the following ACT codes: R03AC, R03AK, R03BA, R03BB, R03CC, R03DA, R03DC, and V03AN01 (oxygen).

**Table 1:** Characteristics of the 34,017 incident lung cancer patients and the 340,170 comparison subjects

	Women				Men				All			
	Lung cancer patients		Comparison subjects		Lung cancer patients		Comparison subjects		Lung cancer patients		Comparison subjects	
Total	15,655	46.0%	156,550	46.0%	18,362	54.0%	183,620	54.0%	34,017		340,170	
Age												
40–52	1317	8.4%	13,129	8.4%	1000	5.5%	10,018	5.5%	2317	6.8%	23,147	6.8%
53–64	4514	28.8%	45,175	28.9%	5001	27.2%	50,099	27.3%	9515	28.0%	95,274	28.0%
65–76	6432	41.1%	64,371	41.1%	8074	44.0%	80,840	44.0%	14,506	42.6%	145,211	42.6%
77–90	3392	21.7%	33,875	21.6%	4287	23.3%	42,663	23.2%	7679	22.6%	76,538	22.6%
Country of origin												
Danish	15,172	96.9%	148,764	95.0%	17,550	95.6%	175,226	95.4%	32,722	96.2%	323,990	95.2%
Immigrant/descendant (Western)	357	2.3%	4348	2.8%	410	2.2%	4115	2.2%	767	2.3%	8464	2.5%
Immigrant/descendant (non‐Western)	126	0.8%	3438	2.2%	402	2.2%	4278	2.4%	528	1.5%	7716	2.3%
Marital status												
Married/cohabitating	6799	43.4%	77,584	49.5%	10,780	58.7%	118,393	64.5%	17,579	51.7%	195,977	57.6%
Living alone	7157	45.7%	61,976	39.6%	5664	30.9%	46,046	25.1%	12,821	37.7%	108,022	31.8%
Unknown	1699	10.9%	16,990	10.9%	1918	10.4%	19,181	10.4%	3617	10.6%	36,171	10.6%
Education												
Basic	9285	59.3%	75,282	48.1%	7942	43.3%	67,866	37.0%	17,227	50.6%	143,148	42.1%
Short	4573	29.2%	53,011	33.9%	8063	43.9%	82,883	45.1%	12,636	37.1%	135,894	40.0%
Long	1267	8.1%	22,220	14.2%	1463	8.0%	25,022	13.6%	2730	8.0%	47,242	13.9%
Unknown	530	3.4%	6037	3.8%	894	4.8%	7849	4.3%	1424	4.3%	13,886	4.0%
Labor market affiliation												
Working	3068	19.6%	37,114	23.7%	3777	20.6%	47,291	25.8%	6845	20.1%	84,405	24.8%
Unemployed	228	1.5%	1862	1.2%	331	1.8%	2036	1.1%	559	1.6%	3898	1.1%
Retirement pension	12,029	76.8%	114,341	73.0%	13,864	75.5%	131,491	71.6%	25,893	76.1%	245,833	72.3%
Other	330	2.1%	3233	2.1%	390	2.1%	2801	1.5%	720	2.2%	6034	1.8%
Income												
Low	3665	23.4%	31,606	20.2%	4374	23.8%	35,190	19.1%	8039	23.6%	66,796	19.6%
Middle	8745	55.9%	78,870	50.4%	10,085	54.9%	89,395	48.7%	18,830	55.4%	168,265	49.5%
High	3245	20.7%	46,074	29.4%	3903	21.3%	59,035	32.2%	7148	21.0%	105,109	30.9%
Charlson comorbidity index score												
0	8874	56.7%	115,787	74.0%	9752	53.1%	124,259	67.7%	18,626	54.8%	240,046	70.6%
1	3544	22.6%	21,910	14.0%	4097	22.3%	29,464	16.0%	7641	22.5%	51,374	15.1%
≥2	3237	20.7%	18,853	12.0%	4513	24.6%	29,897	16.3%	7750	22.7%	48,750	14.3%

#### Lung cancer stage

Information about stage was obtained from DCR and the DLCR. The pTNM was used if available. If the two registries differed in regard to the TNM stage, the DLCR was used. Stage at diagnosis was dichotomized into local and advanced disease. A cut‐point between stage IIB and IIIA was chosen since a previous study has documented a significant difference in mortality between these two stages 21. If any of the T or N values were missing, we categorized SCLC as limited if the tumor was M0 and as extensive if the tumor was M1 regardless of the values, known or unknown, of other components. We categorized NSCLC as advanced if the TNM stage included values of T4, N3, or M1, regardless of other components 22.

#### Characteristics of study population

Demographic and socioeconomic information was collected from Statistics Denmark. This included country of origin categorized into “Danish”, “Immigrant/descendant from a Western country”, or “Immigrant/descendant from a non‐Western country”. Marital status 12 months prior to the diagnosis date was categorized into “living alone” or “cohabitating”. Data on taxable income were extracted for the calendar year preceding the diagnosis date and categorized into three groups using the OECD‐modified scale: “Low” (the lowest 20%), “Middle” (the middle 50%), and “High” (the highest 30%)^23^. The highest attained level of education was categorized into “Basic”, “Short”, “Long”, and “Unknown” according to the international Standard Classification of Education 24. The Charlson Comorbidity Index (CCI) was used to account for comorbidity 25. The CCI was calculated on the basis of diagnoses registered in the NPR in a 10‐year‐period preceding the 12 months prior to the diagnosis date. We grouped the CCI into “Low” (CCI score = 0), “Moderate” (CCI score = 1–2), and “severe” (CCI score ≥3).

### Statistical analysis

Odds ratios (ORs) for having a contact, a radiograph, a lung function test or a medicine prescription were calculated using conditional logistic regression, taking account of the matched design. Unconditional logistic regression analyses were made to compare lung cancer patient with local and advanced stage, and lung cancer patients with and without COPD. A negative binomial regression model applying cluster robust variance at practice level was used to calculate incidence rate ratios (IRRs) for comparisons of monthly rates of contacts, lung function tests, X‐rays, and prescriptions between lung cancer patients and the comparison group in the year before diagnosis.

Analyses were performed separately for each sex because of known differences in the use of general practice among men and women 26, 27. However, the estimates did not differ meaningfully between men and women, and are therefore presented combined (See appendix, Table 2 and Table 3, for separate analyses).

All analyses were adjusted for socioeconomic, demographic variables, age, and comorbidity. Data were analyzed using the statistical software Stata 13.0 (StataCorp LP, Texas, TX).

### Ethics

The study was approved by the Danish Data Protection Agency (j.no. 2009‐41‐3471).

## Results

The study included 34,017 lung cancer patients and 340,170 comparison subjects. The study subjects were listed with 2676 general practices. Among the lung cancer patients 18.6% had localized disease (stage IA–IIB), 81.4% had advanced disease (stage IIIA–IV), and 0.7% of cases had no information on stage. A total of 7551 lung cancer patients (22.2%) were identified as having COPD compared to 11.9% in the comparison group.

The patients and comparison subjects differed according to socio‐demographic variables and comorbidity (Table 1). Lung cancer patients were more likely to be living alone and had lower income, lower education level, and higher comorbidity score. The mean age at diagnosis was 68 and 69 years for women and men, respectively.

**Figure 1:**
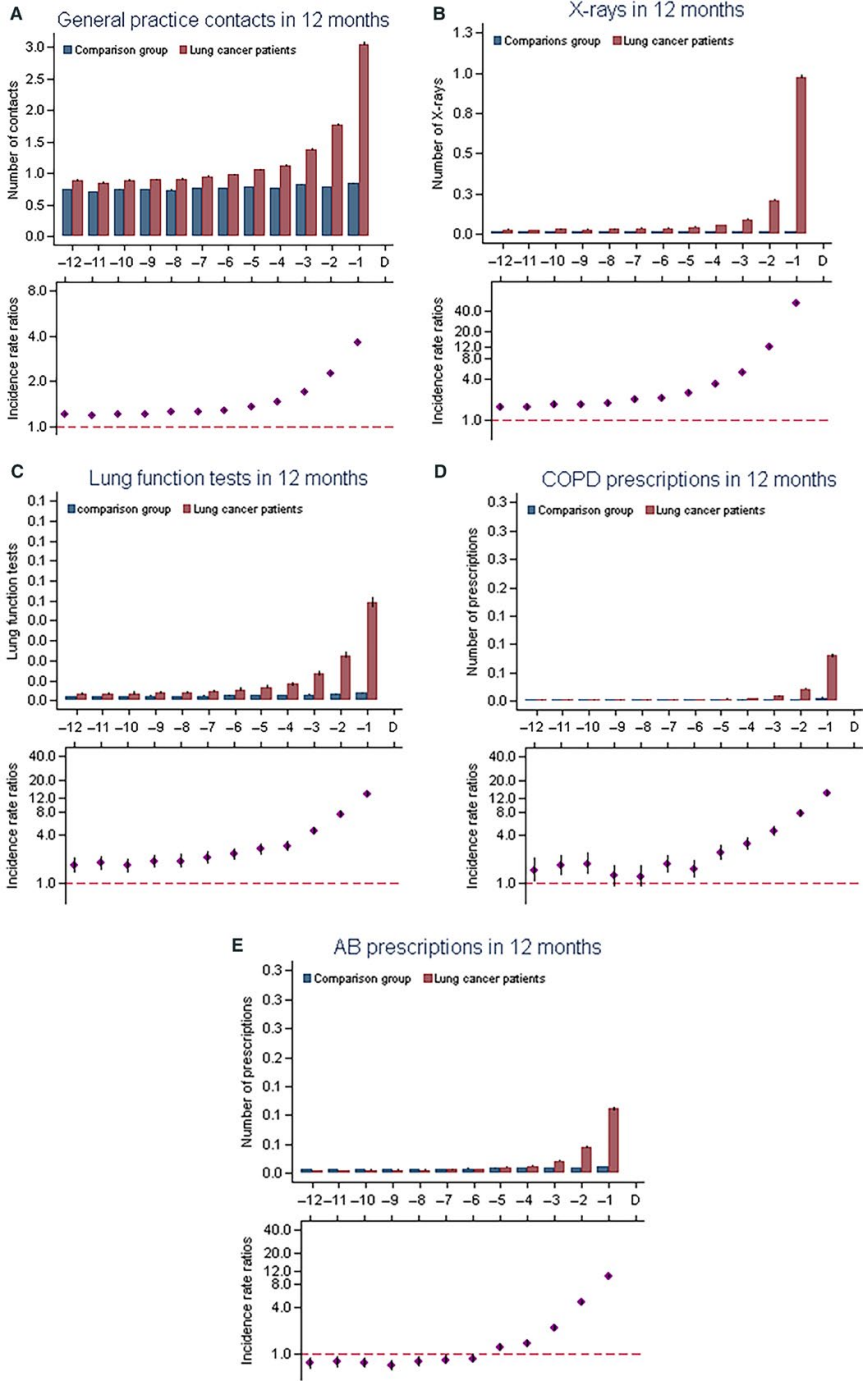
Contacts (A), X‐rays (B), lung function tests (C), chronic obstructive pulmonary disease (COPD) prescriptions (D), and antibiotics prescriptions (E) in general practice. Upper part: Mean numbers of contacts, lung function test, X‐rays, or prescriptions in general practice for lung cancer patients and matched comparison subjects prior to diagnosis/date with 95% confidence interval. Lower part: the incidence rate ratio (IRR) with 95% CIs.

During months 12 to 1 prior to diagnosis (i.e., omitting the last month before diagnosis), 92.6% of lung cancer patients and 88.4% of comparison subjects had one or more contacts to general practice (Table 2). The odds for having nine or more contacts were higher among lung cancers patients compared to comparison subjects (OR: 1.89 [95 CI: 1.83–1.94]) (Table 2). Lung cancer patients had significantly higher frequencies of GP contacts from four months prior to diagnosis and the differences rose consistently with a peak in the last month before diagnosis (Fig. 1A).

**Table 2:** Proportion of persons with consultations, lung function tests, radiographs, Antibiotics prescriptions, and COPD medicine prescriptions during months 12 to 1 prior to lung cancer diagnosis

	ALL study subjects					All lung cancers				
	Lung cancer patients		Comparison subjects		OR (95%CI)^a^	Localized		Metastatic		OR (95% CI)^a^
Consultations										
0	2507	7.4%	46399	13.6%	0.42 (0.39–0.44)	544	8.7%	1903	6.9%	0.74 (0.65–0.83)
1–4	6980	20.5%	98198	28.9%	0.68 (0.65–0.70)	1244	19.8%	5659	20.6%	0.99 (0.91–1.07)
5–8	6889	20.3%	72491	21.3%	0.98 (0.95–1.01)	1250	19.9%	5602	20.4%	1.00 (0.93–1.08)
≥9	17648	51.8%	123082	36.2%	1.89 (1.83–1.94)	3232	51.6%	14332	52.1%	1.09 (1.02–1.16)
Lung function tests										
0	31500	92.6%	331855	97.5%	0.29 (0.27–0.30)	5787	92.3%	25473	92.6%	1.06 (0.94–1.18)
1	2342	6.9%	7778	2.3%	3.44 (3.26–3.63)	451	7.2%	1880	6.8%	0.94 (0.84–1.06)
≥2	175	0.5%	537	0.2%	3.74 (3.08–4.55)	32	0.5%	143	0.6%	1.00 (0.66–1.52)
Radiographs										
0	23361	68.7%	300836	88.4%	0.28 (0.27–0.29)	3637	58.0%	19564	71.2%	1.89 (1.78–2.01)
1	6223	18.3%	28051	8.3%	2.43 (2.35–2.52)	1443	23.0%	4734	17.2%	0.70 (0.65–0.75)
≥2	4433	13.0%	11283	3.3%	4.26 (4.08–4.45)	1190	19.0%	3198	11.6%	0.54 (0.50–0.59)
Antibiotics										
0	19410	57.1%	258900	76.1%	0.42 (0.41–0.43)	3345	53.4%	15919	57.9%	1.20 (1.13–1.28)
1	7527	22.1%	52412	15.4%	1.54 (1.50–1.59)	1486	23.6%	5991	21.8%	0.90 (0.84–0.97)
≥2	7080	20.8%	28858	8.5%	2.70 (2.61–2.78)	1439	23.0%	5586	20.3%	0.86 (0.80–0.92)
COPD prescriptions										
0	32462	95.4%	335382	98.6%	0.30 (0.28–0.32)	5979	95.4%	26239	95.4%	1.01 (0.87–1.16)
≥1	1555	4.6%	4788	1.4%	3.33 (3.12–3.55)	291	4.6%	1257	4.6%	0.99 (0.86–1.15)

OR, odds ratio; CI, confidence interval; CCI, Charlson Comorbidity Index; COPD, chronic obstructive pulmonary disease.

a Adjusted analyses comparing the activity between lung cancer patients and comparison subjects. ^2^Adjusted analyses comparing the activity between lung cancer patients with localized disease and lung cancer patients with metastatic disease. ORs are adjusted for age, county of origin, marital status, education, labor market affiliation, income, and comorbidity (CCI).

251 patients without information about stage at diagnosis are omitted.

13.0% of the lung cancer patients and 3.3% of the comparisons had two or more radiographs performed during the 11 months before the last month before diagnosis (OR: 4.26 [95% CI: 4.08–4.45]) (Table 2). The rates were higher for lung cancer patients throughout all 12 months before diagnosis with a large excess just before diagnosis (Fig. 1B).

7.4% of the lung cancer patients and 2.5% of comparison subjects had one or more first‐time lung function test performed in general practice during the 11 months before diagnosis. During the 12 months before diagnosis the number of lung function test were significantly higher among lung cancer patients than among the comparisons (Fig. 1C) and the rates rose until peaking in the last month (IRR: 13.1 [95% CI: 12.2–14.2]).

During the last 11 months before the month before diagnosis, 20.8% of the lung cancer patients and 8.5% of the comparisons persons were treated with antibiotics twice or more (OR: 2.70 [95% CI: 2.61–2.78]) (Table 2). The higher number of antibiotic prescriptions was mainly seen in the last 4 months before diagnosis (Fig. 1E), during month 12 to 6 prior to diagnosis lung cancer patients were treated less with antibiotics than compared to the comparison subjects.

Furthermore, 4.6% of the lung cancer cases and 1.4% of the comparisons had one or more new prescription of COPD medicine (Table 2).

### Lung cancer patients with localized disease versus advanced disease

8, 7% of lung cancer patients with localized disease had no contacts in general practice compared to 6.9% of patients with advanced disease (OR: 0.74 [95% CI: 0.65–0.83]) (Table 2) whereas the odds for having more contacts were similar between the two groups. 19.0% of patients with localized disease had two or more radiographs performed during the 11 months before diagnosis compared to 11.6% of patients with advanced disease (OR = 0.54 [95% CI: 0.50–0.59]). The use of lung function test in general practice and COPD prescriptions did not differ between patients with localized disease and patients with advanced disease, however, 23.0% of patients with localized disease and 20.3% of patients with advanced disease had two or more prescriptions of antibiotics (OR: 0.86 [95% CI: 0.80–0.92]).

### Lung cancer patients with COPD versus other lung cancer patients

Lung cancer patients with COPD had more contacts in the 11 months prior to diagnosis than lung cancer patients without COPD (OR: 2.06 [95%CI: 1.93–2.19]) and they also had higher odds of having lung function tests performed. Lung cancer patients with COPD had more contacts (≥9: OR: 1.52 [95% CI: 1.43–1.62]), more lung function tests (≥2 OR: 3.23 [95% CI: 2.67–3.80]), more X‐rays (≥2 OR: 2.23 [95% CI: 2.07–2.40]) and more prescriptions of antibiotics (≥2 OR: 1.93 [95% CI: 1.82–2.05]) than other COPD patients (Table 3). The difference in diagnostic activity was higher when comparing lung cancer patients and comparison subjects without COPD than when comparing lung cancer patients and comparisons diagnosed with COPD. 11% of lung cancer patients without COPD and 2.6% of comparisons without COPD had two or more X‐rays performed 12 to 1 months before diagnosis (OR: 4.19 [95% CI: 3.98–4.41]).

**Table 3:** Proportion of patients with consultations, lung function tests, radiographs, Antibiotics prescriptions during months 12 to 1 prior lung cancer diagnosis

	All lung cancers					with COPD					without COPD				
	COPD		No COPD		OR (95%CI)^a^	Lung cancer patients		Comparison subjects		OR (95% CI)^a^	Lung cancer patients		Comparison subjects		OR (95% CI)^a^
Consultations															
0	178	2.4%	2329	8.8%	0.35 (0.29–0.42)	178	2.4%	1313	4.0%	0.63 (0.52–0.76)	2329	8.8%	40083	14.6%	0.49 (0.46–0.52)
1–4	799	10.6%	6181	23.4%	0.50 (0.46–0.55)	799	10.6%	5478	16.6%	0.63 (0.58–0.69)	6181	23.4%	83155	30.3%	0.73 (0.70–0.75)
5–8	1217	16.1%	5672	21.4%	0.78 (0.72–0.84)	1217	16.1%	6554	20.0%	0.79 (0.73–0.85)	5672	21.4%	59135	21.5%	1.06 (1.02–1.80)
≥9	5359	70.9%	12284	46.4%	2.06 (1.93–2.19)	5359	70.9%	19510	59.4%	1.52 (1.43–1.62)	12284	46.4%	92229	33.6%	1.74 (1.69–1.80)
Lung function tests															
0	6624	87.7%	24605	93.0%	0.40 (0.39–0.46)	6624	87.7%	31384	95.5%	0.34 (0.32–0.37)	24605	93.0%	263397	95.9%	0.85 (0.80–0.90)
1	612	8.1%	1730	6.5%	1.29 (1.16–1.44)	612	8.1%	1026	3.1%	2.69 (2.40–3.01)	1730	6.5%	7680	2.8%	1.45 (1.31–1.59)
≥2	315	4,1%	131	0.5%	6.35 (5.92–6.99)	315	4.2%	445	1.4%	3.23 (2.67–3.80)	131	0.5%	3525	1.3%	0.50 (0.36–0.65)
Radiographs															
0	4384	58.0%	18977	71.7%	0.71 (0.67–0.75)	4384	58.0%	24483	74.5%	0.48 (0.46–0.51)	18977	71.7%	247448	90.1%	0.27 (0.26–0.28)
1	1635	21.7%	4588	17.3%	1.15 (1.07–1.23)	1634	21.7%	5218	15.9%	1.37 (1.28–1.46)	4588	17.3%	20040	7.3%	2.69 (2.59–2.80)
≥2	1532	20.3%	2901	11.0%	1.49 (1.39–1.61)	1532	20.3%	3154	9.6%	2.23 (2.07–2.40)	2901	11.0%	7114	2.6%	4.19 (3.98–4.41)
Antibiotics															
0	2973	39.4%	16437	62.1%	0.42 (0.39–0.44)	2973	39.4%	18177	55.3%	0.53 (0.50–0.57)	16437	62.1%	215667	78.5%	0.45 (0.43–0.46)
1	1784	23.6%	5743	21.7%	1.08 (1.01–1.16)	1784	23.6%	7289	22.2%	1.05 (0.99–1.13)	5743	21.7%	40174	14.6%	1.62 (1.57–1.68)
≥2	2794	37.0%	4286	16.2%	2.85 (2.67–3.04)	2794	37.0%	7389	22.5%	1.93 (1.82–2.05)	4286	16.2%	18761	6.8%	2.59 (2.48–2.69)

OR, odds ratio; CI, confidence interval; COPD, chronic obstructive pulmonary disease.

a Adjusted analyses comparing the activity between patients with COPD and patients without COPD (first set of columns) and comparing activity between lung cancer patients and comparison subjects (the next two sets of columns). ORs are adjusted for age, county of origin, marital status, education, labor market affiliation, income, and comorbidity (CCI).

In order to estimate the clinical relevance of the observed excess of general practice use we calculated risk estimates in the form of positive predictive values (PPVs). We calculated PPVs for having ≥9 contacts, ≥1 lung function test, ≥2 X‐rays, and ≥2 medicine prescriptions, also stratified by COPD. All PPVs calculated were below 1% (0.3% for ≥9 contacts to 0.9% for ≥2 X‐rays). These low PPVs are in range with many symptoms for lung cancer 11.

## Discussion

This study, including 34,017 Danish lung cancer patients, found a higher frequency of contacts, diagnostic tests, and prescriptions in general practice for lung cancer patients compared with a comparison group, with a steep increase before the month prior to diagnosis. There was no important difference in activity when dividing the lung cancer patients into localized and advanced disease at diagnosis which indicates that they might present with similar symptoms although patients with advanced lung cancer had fewer radiographs before diagnosis than patients with local cancers. When dividing the study group according to COPD, the difference in activity between lung cancer patients and comparisons where more pronounced in the group without COPD. This indicates that having COPD can mask symptoms of lung cancer.

### Strengths and limitations

The strength of this study was a high statistical precision owing to the large, national study population. All data were collected from nationwide Danish registers. Cancer patient data were included from The Danish Cancer Registry and the Danish Lung Cancer Registry in order to have an almost complete inclusion of patients and clinical data 15, 16, 17. The information on healthcare services provided in primary care is considered to be valid as registration of these services forms the basis for remuneration of the GPs 17. By matching lung cancer patients and comparison persons according to age, gender, and general practice we diminished the risk of confounding. However, the results may have been influenced by confounding by smoking as we were not able to obtain smoking history, and there may be residual confounding by comorbidity as CCI does not include diseases managed only in general practice.

In the analyses of general practice activity we omitted the last month before diagnosis to eliminate the inevitable increase in activity just before diagnosis. Furthermore, we omitted patients' lung function tests and prescriptions if they had such tests in the months between 12 and 36 before diagnosis. Test and prescriptions included in the analyses where therefore first‐time, reflecting the GPs response to the symptoms of the patients seen in practice at that time. Thus we predominantly explored new episodes of symptoms or signs where the GPs response would be a lung function test or prescription of medicine.

A limitation of the study was the lack of information on other lung diseases such as asthma, these diseases will increase GP contact rate and would probably be uneven distributed with a higher incidence among patients with lung cancer. However, a large proportion of these patients would have been in an ongoing follow‐up for the disease and they would have had lung function tests and prescriptions for medication in years before diagnosis. These tests would therefore have been omitted in the analyses. A further limitation of this study was a lack of information on the reasons for the consultations with the GPs.

We restricted our analyses to first occurrence of cancer to avoid the influence of an increased awareness of the GP among patients with a history of cancer. Being population based and thus including all lung cancer patients make the findings generalizable to other relevant health care systems.

Excess consultations among the lung cancer patients was also found in Danish study mapping the routes to diagnosis of lung cancer patients 12 and a UK study of consultations before diagnosis 28.

The fact that lung cancer patients are seen in general practice before diagnosis and that the GP acts on the contacts with a high range of treatment and diagnostic activity months before diagnosis (even when omitting the last month) can be based on two things (or both things combined); either the GPs suspects cancer but fails to make the one diagnostic test to find the cancer (or fails to refer timely to fast‐track) or the GP do not suspect cancer by interpreting the symptoms as something else. The latter is supported by the increased use of lung function tests, antibiotics, and COPD medication. A Danish study found that comorbidity delayed diagnosis in around 23% of lung cancer patients seen by GPs 29. Symptoms like cough, weight loss, or breathlessness may be ascribed to known comorbidity rather than lung cancer. This is in line with our findings.

Many of the lung cancer patients had two or more X‐rays performed prior to diagnosis which is in line with results from a Danish study mapping the routes to diagnosis 12. For patients with localized disease this excess of X‐rays was more pronounced. This could be based on false negative X‐rays which occur in as much as a quarter of cancer patients 30, 31.

Patients with advanced disease had more contacts to general practice than patients with localized disease. This could be patients, with localized disease, bypassing general practice, and getting the diagnosis in connection with a hospital inpatients visit for other diseases and thus having an earlier lung cancer diagnosis.

We found virtually no difference in activity between patients with localized disease and advanced disease. A British study from 2015 found no difference in symptoms presented in general practice according to stage at diagnosis 32. However, the difference we did find (more X‐rays in the group of patients with localized disease) may be rooted in the X‐rays poor sensitivity, especially for small tumors.

The results, on the other hand, indicate an opportunity to optimize the early detection of lung cancer in general practice. So the question remains, how to select patients for further examination. GP awareness could be relevant as a Danish study from 2014 suggests that providing GPs with a short update on lung cancer (early symptoms, how to refer timely) would make the GPs refer more patients to examinations 33. Earlier and faster diagnosis may also be achieved by granting GPs free, direct access to low‐dose computed tomography; this would provide them with a more sensitive test for lung cancer than the X‐rays.

## Conclusion

The findings of this large population‐based study revealed an increase in healthcare seeking and diagnostic activity in general practice among lung cancer patients prior to their diagnosis. This was not modified by the stage of the cancer but by whether the patients had COPD or not. Overall, the results indicate that a “diagnostic time window” is present, in which there is opportunity to diagnose lung cancer more timely in general practice. The high number of repeated X‐rays, lung function tests, and prescription of lung medicine and antibiotics show that GPs start investigating the patient's months before the diagnosis. The results support Continuing Medical Education for GPs and indicate a need for more studies on how to select patients for further diagnostic test and which test may be the most optimal in general practice.

## Conflict of Interest

None declared.
